# Outcomes of Total Hip Arthroplasty for Congenital Hip Dislocation: A Retrospective Study

**DOI:** 10.7759/cureus.84797

**Published:** 2025-05-25

**Authors:** Mohamed-Anas Zeroual, Mohamed Nassiri, Mostapha El Kasseh, Abdessalam Achkoun, Rachid Chafik

**Affiliations:** 1 Orthopedics and Traumatology Department A, Ibn Tofail Hospital, Centre Hospitalier Universitaire (CHU) Mohammed VI, Faculté de Médecine et de Pharmacie de Marrakech, Marrakesh, MAR

**Keywords:** congenital dislocation of the hip, developmental dysplasia of the hip (ddh), dysplasia, morocco, total hip arthroplasty (tha)

## Abstract

Introduction

Congenital hip dislocation (CHD) represents a long-standing public health issue in Morocco, with a late diagnosis often leading to advanced joint degeneration. While periacetabular osteotomy is preferred in young adults with preserved joint space, total hip arthroplasty (THA) becomes necessary in end-stage cases. However, THA poses challenges due to anatomical deformities and the increased likelihood of revision surgeries. This study evaluates the clinical, radiological, and functional outcomes of THA performed for CHD and compares them with existing literature.

Materials and methods

A retrospective study was conducted on 16 patients (19 hips) who underwent THA for CHD between 2008 and 2022 at a single orthopedic unit. Patients aged over 15 years with a minimum follow-up of 18 months were included. Clinical assessment using the Postel and Merle d'Aubigné score (PMA), radiological classification using modified Cochin classification (Co), and postoperative complications were analyzed.

Results

The mean age was 35.1 years, with a female predominance (68.75%). High dislocations (fixed or non-fixed) represented the majority of cases (75%). Preoperatively, the average PMA score was 8.41/18. Lameness was present in 87.5% of cases, and leg length discrepancies (LLDs) were significant, with an average of 39.25 mm, especially in high dislocations. All THAs used cemented components, with the use of bone grafts in three cases (18.75%) and release techniques in six cases (37.5%). The average follow-up period for patients was 22 months. At the final assessment, the mean PMA score postoperatively improved to 17.23/18. Complications included intraoperative fractures in three cases (18.75%), nerve injury in two cases (12.5%), dislocation in one case (6.25%), and trochanteric non-union in one case (6.25%).

Conclusion

THA in CHD can achieve satisfactory mid-term functional outcomes when performed with meticulous preoperative evaluation and adapted surgical techniques. However, the procedure requires significant surgical expertise due to its complexity and potential complications.

## Introduction

Congenital hip dislocation (CHD) includes a variety of anomalies ranging from acetabular dysplasia to complete dislocation of the femoral head from the acetabulum. In Morocco, CHD remains a significant public health issue, with an estimated incidence of five to ten per 1000 births [1]. This high prevalence is mainly due to limitations in neonatal screening and access to early treatment, which often results in delayed diagnosis and management [2,3].

Left untreated, CHD leads to significant functional disability, gait abnormalities, and early osteoarthritis. When conservative procedures are no longer viable, total hip arthroplasty (THA) becomes the only solution to restore mobility and relieve pain [4]. However, performing THA in patients with CHD is technically demanding due to the complex anatomical alterations such as shallow acetabulum, increased femoral anteversion, high-riding dislocations, leg length discrepancy (LLD), and soft tissue contractures [5,6]. These considerations often need complex surgical techniques, such as femoral shortening osteotomies, bone grafts, modular implants, and customized approaches [7].

Various systems have been proposed to classify CHD, including Crowe’s classification [8], Hartofilakidis [5], and the modified Cochin (Co) classification used by the French Society of Orthopaedic and Trauma Surgery (SoFCOT) [9], each offering advantages in preoperative planning and prognosis assessment [10].

Even with all the progress in implants and surgical techniques, THA in CHD patients still poses a higher risk of complications, including intraoperative fractures, nerve damage, implant loosening, and postoperative instability, particularly in a younger and demanding population [11]. However, multiple studies have demonstrated that with thorough preoperative evaluation, personalized surgical planning, and adequate rehabilitation, positive mid- and long-term outcomes can be achieved [12,13].

This study aims to evaluate the clinical, radiological, and functional outcomes of THA performed for CHD in a Moroccan population and to compare these findings with the existing literature. By analyzing a series of patients with varying dislocation degrees treated over a 14-year period, this research seeks to contribute to the understanding and optimization of THA strategies in this challenging pathology.

## Materials and methods

This is a retrospective study of 16 patients who underwent surgery at the Orthopedics and Traumatology Department A of Ibn Tofail Hospital in Marrakech (Morocco) between 2008 and 2022. We included all patients over 15 years of age with a complete operative file and a minimum follow-up of 18 months. The data analysis was performed using IBM SPSS Statistics for Windows, Version 25 (Released 2017; IBM Corp., Armonk, New York, United States). Epidemiological data were collected (age, sex, unilateral or bilateral involvement) along with data regarding previous surgical history and functional signs (pain, lameness, associated signs). Patients underwent clinical evaluation using the Postel and Merle d'Aubigné (PMA) score, assessing pain, mobility, and ability to walk, each rated from one (worst condition) to six (best condition). Scores range from a minimum total of three to a maximum of 18. This evaluation, together with radiological analysis, was performed both preoperatively and postoperatively. Patients were then classified according to the modified Co classification system, which defines five grades based on femoral head position: from centered in the acetabulum dysplasia (Grade 1) to subluxation (Grade 2), low anterior dislocation (Grade 3), high fixed intermediate dislocation (Grade 4), and high non-fixed posterior dislocation (Grade 5) [9,14]. We recorded the different approaches used, as well as intraoperative and postoperative early and late complications. A clinical and radiological study was carried out at follow-up, focusing on the different clinical aspects (PMA, lameness, LLD) and radiological aspects (consolidation and signs of dislocation).

## Results

**Figure 1:**
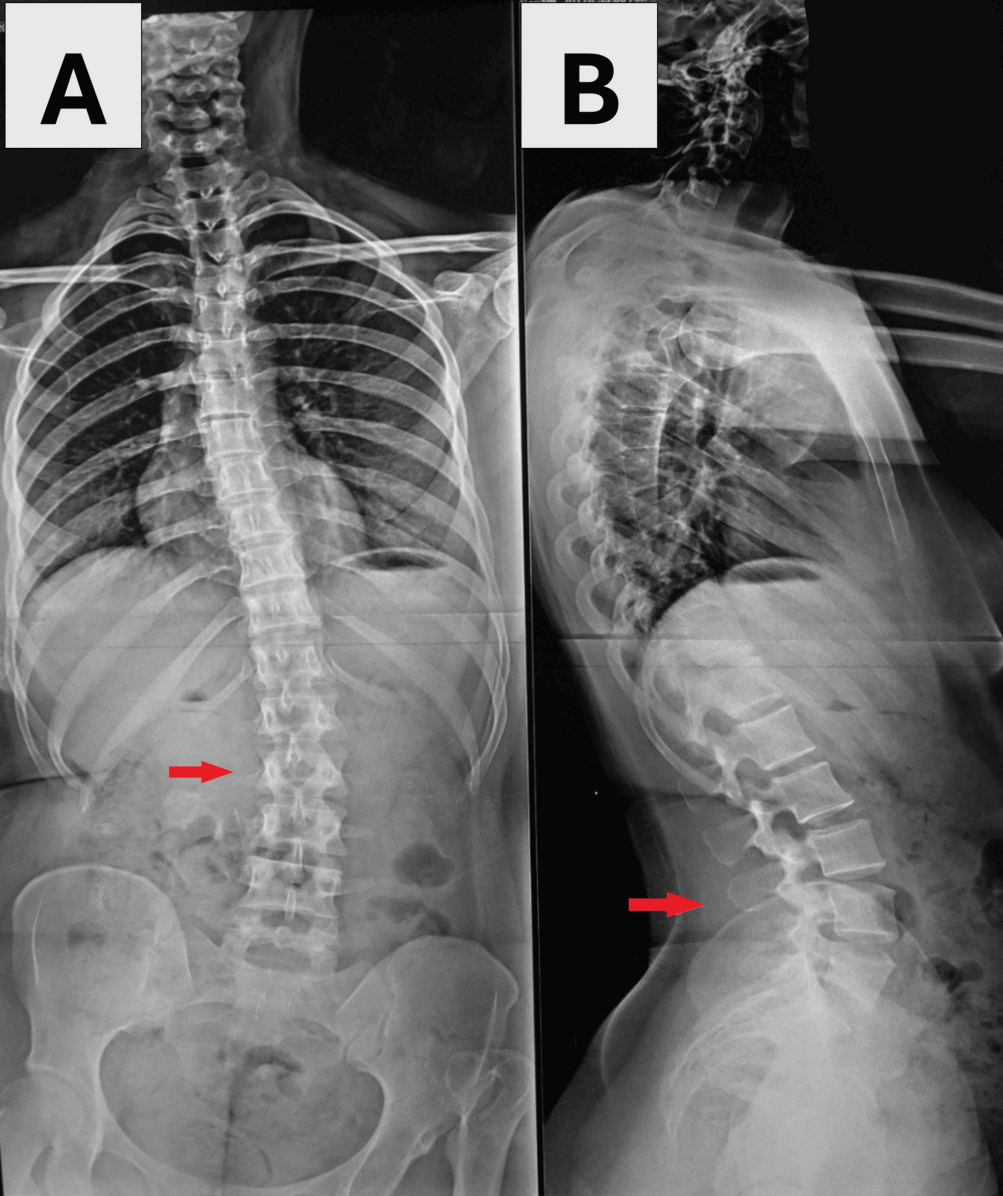
Anteroposterior and lateral radiographs of a 42-year-old female patient in our study A, B: left congenital hip dislocation, suffering from severe scoliosis (red arrows)

**Figure 2:**
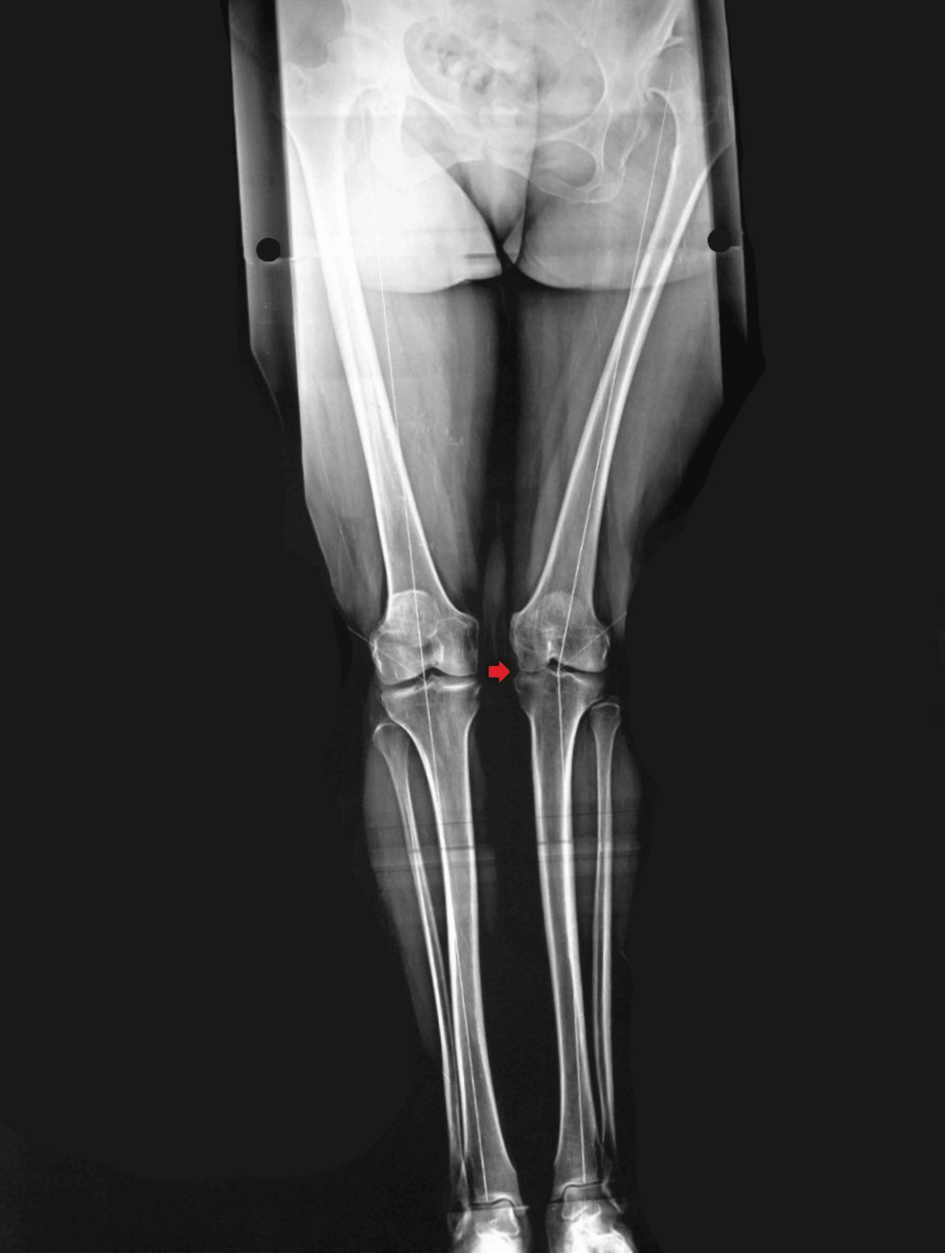
Pangonogram of a 36-year-old female patient in our study She was suffering from left congenital hip dislocation, showing a 13° valgus deformity, leading to a significant destruction of the medial compartment of the left knee (red arrow)

The mean age at surgery was 35.1 years, with extremes ranging from 21 to 58 years, and a clear female predominance was noted, with 68.75% of patients being women (11 females and five males). The right hip was affected in seven cases (43.75%), the left in six cases (37.5%), and bilateral involvement in three cases (18.75%). A history of prior hip surgery was reported in two of our patients (12.5%). The primary complaint leading to consultation was pain and limping. Two patients had scoliosis (12.5%) (Figure 1), and two others had gonarthrosis of the left knee (12.5%) (Figure 2).

Of the 16 patients in our series, we performed 19 THAs. The cases were classified using the modified Co classification system (Table 1), which includes five types. Among these, there were seven cases of high-fixed dislocations, which accounted for eight THAs (six unilateral and one bilateral). Three cases of low dislocations, one case of dysplasia, and additionally five cases of high non-fixed dislocations (six unilateral THAs and one bilateral THA). It is worth noting that no cases of subluxation were observed in our series.

**Table 1:** Summary of the different types of congenital hip dislocations, cases per type, total hip arthroplasties per type, and laterality in our study THA: total hip arthroplasty

Types of dislocations	Cases per type	THAs per type	Laterality
Dysplasia	1	1	One unilateral
High fixed dislocation (intermediate)	7	8	Six unilateral, one bilateral
High non-fixed dislocation (posterior)	5	6	Four unilateral, one bilateral
Low dislocation (anterior)	3	4	Two unilateral, one bilateral

LLD averaged 39.25 mm across the series, with an average of 65 mm for high-fixed dislocations (Table 2).

**Table 2:** Summary of average leg length discrepancy by type of congenital hip dislocations in our study LLD: leg length discrepancy

Types of dislocations	LLD average
Dysplasia	12 mm
High non-fixed dislocation (posterior)	62 mm
High fixed dislocation (intermediate)	65 mm
Low (anterior) dislocation	18 mm
The series	39.25 mm

Lameness was present in 87.5% of patients (14 of the 16 patients), so of the 19 hips operated 16 of them had lameness before surgery. The rate was 100% for high dislocations, whether supported or not (Table 3).

**Table 3:** Summary of the lameness rates by type of congenital hip dislocations in our study CHD: congenital hip dislocation

Types of dislocations	Number of cases	Hips with CHD	CHDs with lameness	Lameness rate of total cases
Dysplasia	1	1	1	100%
High non-fixed dislocation (posterior)	5	6 (one bilateral)	6	100%
High fixed dislocation (intermediate)	7	8 (one bilateral)	8	100%
Low dislocation (anterior)	3	4 (one bilateral)	1	33.33%
The series	16	19	16	87.5%

The PMA score was calculated according to the type of dislocation; the average score for our 16 patients was 8.41/18 (Table 4).

**Table 4:** Summary of the Postel and Merle d'Aubigné score by type of congenital hip dislocation before surgery The Postel and Merle d'Aubigné score was adapted in our study, as described in previous publications [14]

Types of dislocations	Pain	Mobility	Ability to walk	Total PMA score
Dysplasia	2.88	2.70	2.76	8.34/18
High non-fixed dislocation (posterior)	2.98	2.46	2.86	8.3/18
High fixed dislocation (intermediate)	3.01	2.34	2.67	8.02/18
Low dislocation (anterior)	3.12	3	2.87	8.99/18
The series	3.03	2.6	2.8	8.41/18

**Figure 3:**
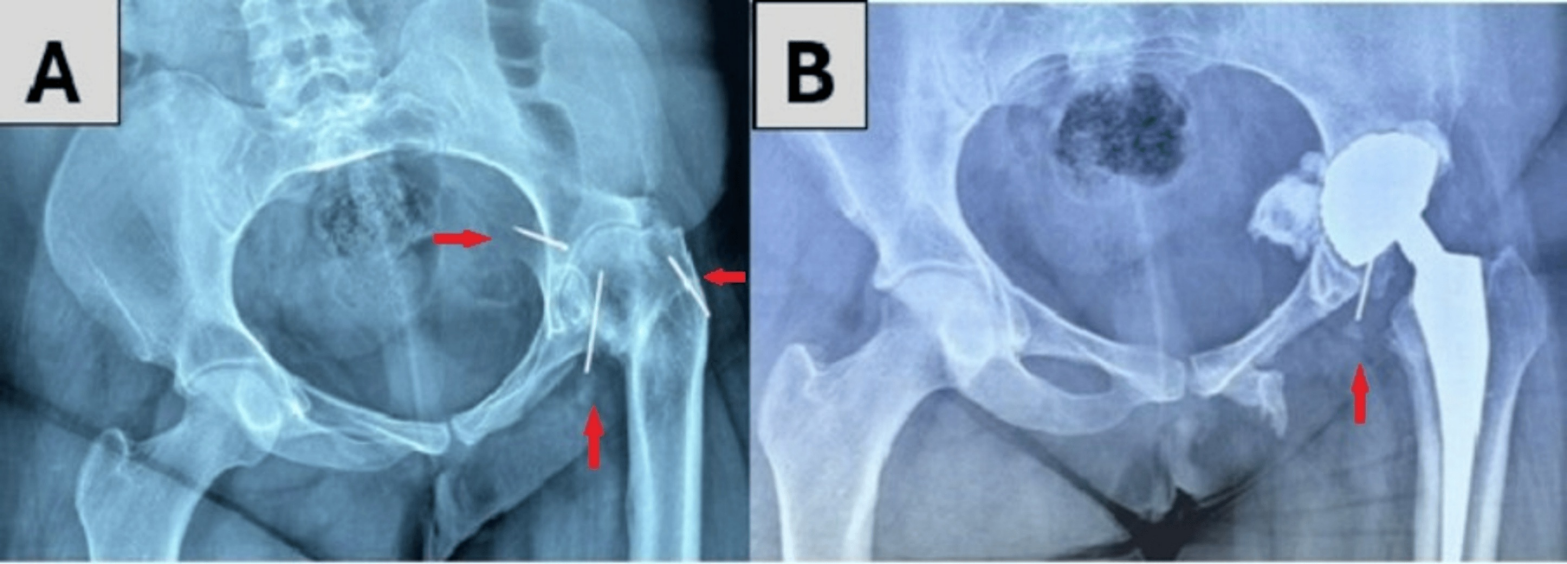
Radiographs of a 35-year-old female in our study, suffering from hip dysplasia with a history of hip surgery and residual pins A: preoperative anteroposterior pelvic radiograph, showing residual pins from a previous childhood hip surgery (red arrows); B: postoperative anteroposterior pelvic radiograph, showing the total hip replacement with a residual pin that proved difficult to remove (red arrow)

The two surgical approaches we used were Hardinge and Moore [15,16]. A trochanterotomy was performed in five patients, representing 31% of all our cases. One patient suffering from acetabular dysplasia had a history of hip surgery with residual pins, which we attempted to remove during THA surgery (Figure 3).

All acetabular cups were cemented at the level of the paleo-acetabulum, with a bone graft performed in three patients and a bone buttress in one patient. We reamed the femur in four patients (25%), and we opted for a dysplastic stem in four patients (25%). A psoas tenotomy was performed in six patients (37.5%) as a release procedure.

**Figure 4:**
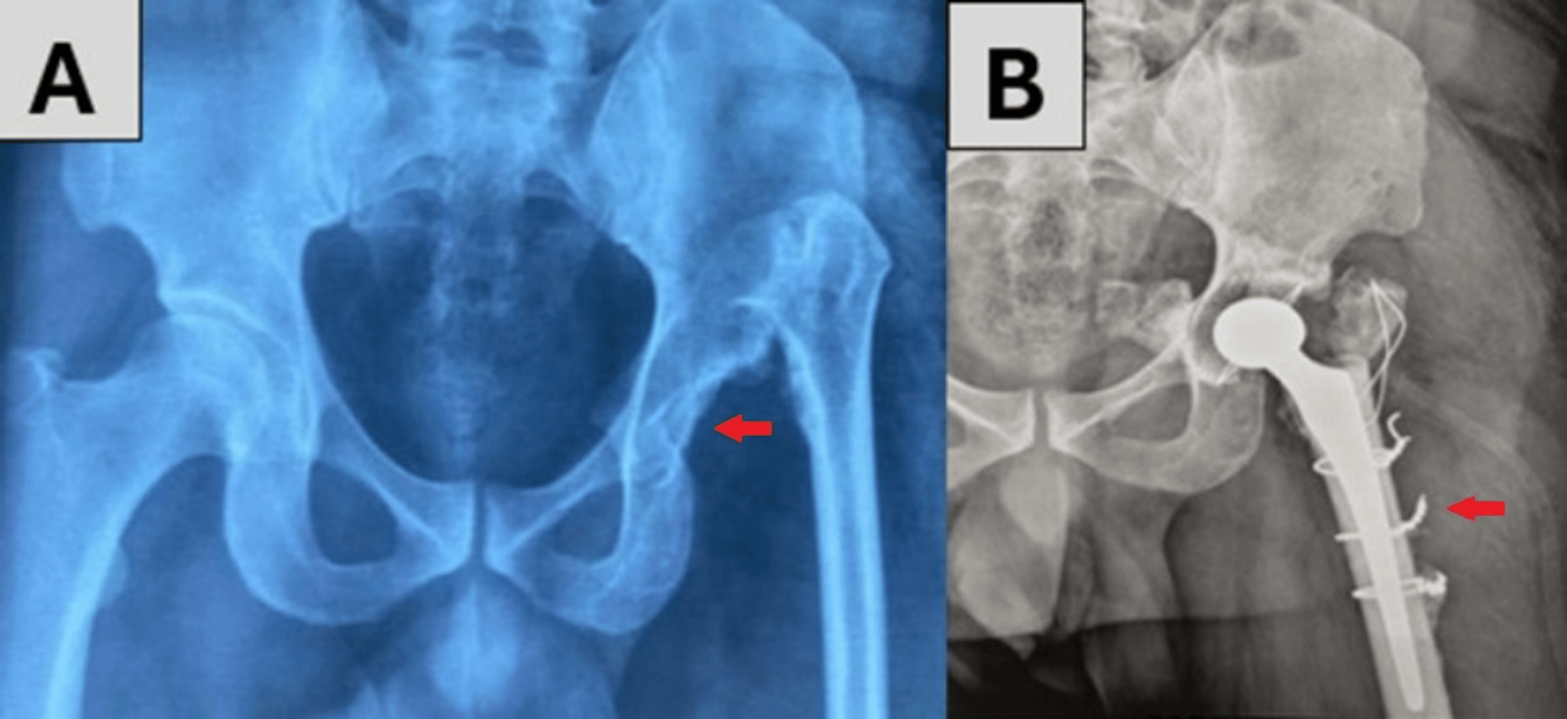
Radiographs of a 42-year-old male in our study, suffering from left congenital hip dislocation She underwent a trochanterotomy procedure complicated by an intra-operative subtrochanteric fracture, which was treated with tension band wiring. A: preoperative anteroposterior pelvic radiograph, showing a left congenital hip dislocation (CHD) with a vacant acetabulum (red arrow); B: postoperative anteroposterior left hip radiograph, showing the total hip arthroplasty with tension band wiring (red arrow)

**Figure 5:**
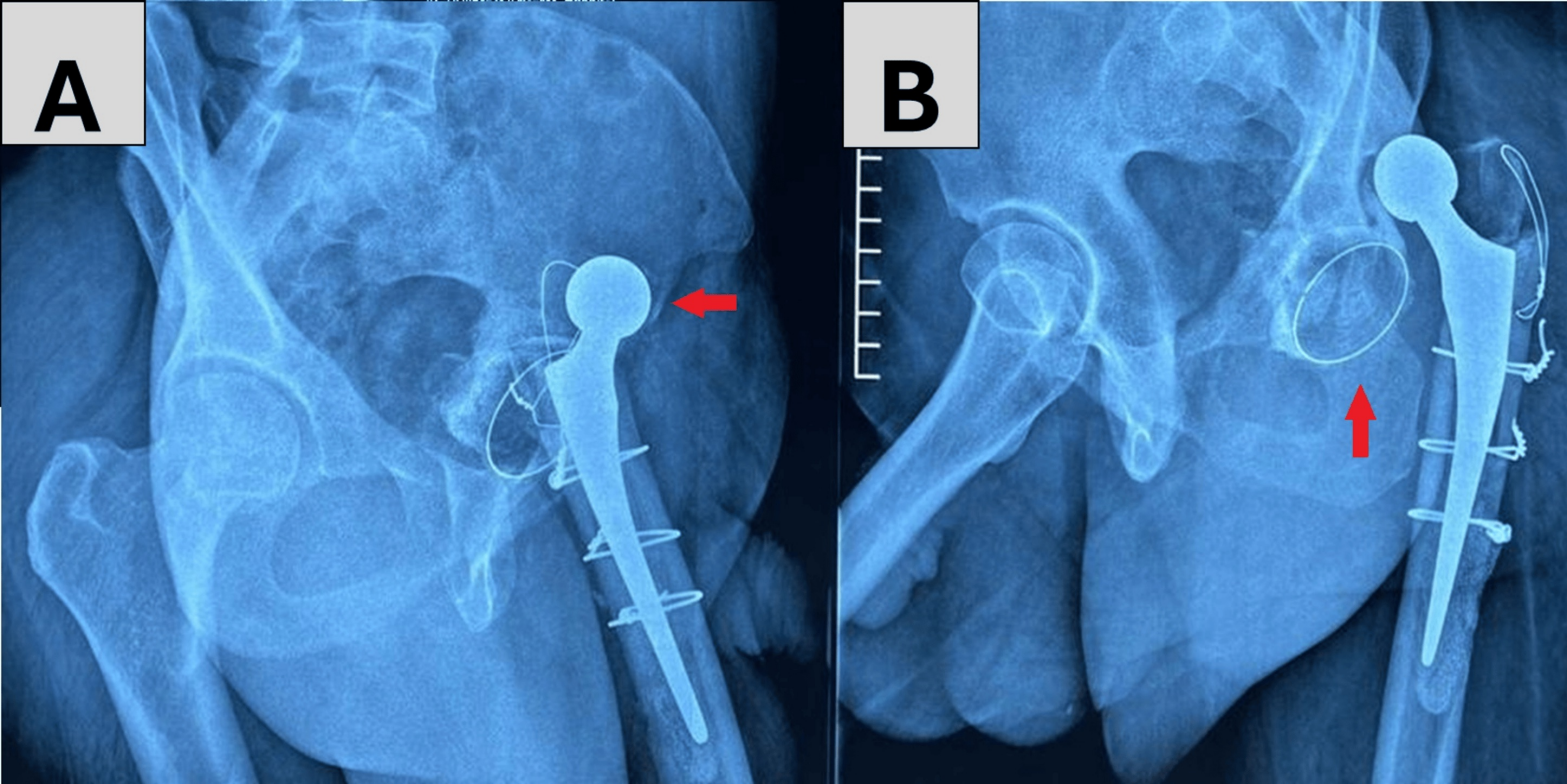
Radiographs of the same patient in Figure 4 during their hospital stay A, B: showing a post-operative dislocation of total hip replacement (red arrows)

**Figure 6:**
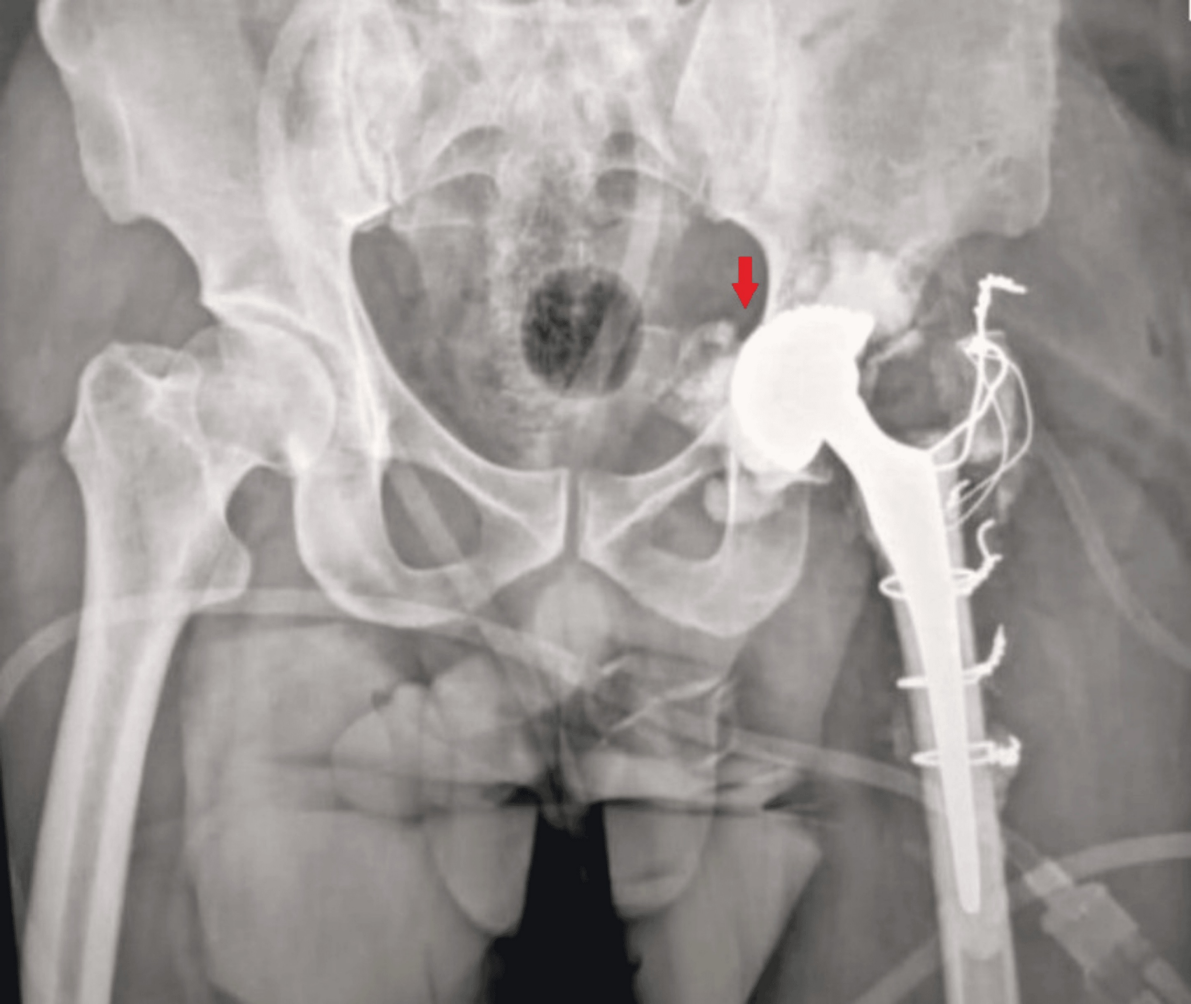
Radiograph following revision surgery with dual mobility total hip arthroplasty (red arrow), of the same patient in Figure 4 and Figure 5

Regarding complications, we encountered three cases of peroperative fractures (18.75%) with a subtrochanteric split, which we treated with tension band wiring (Figure 4), and two cases of nerve paralysis: sciatic and femoral. One case of postoperative dislocation (Figure 5), which was corrected by the placement of a dual mobility THA (Figure 6), and one case of trochanteric non-union.

After an average follow-up of 22 months, an improvement in the PMA score was observed in all our patients with a mean postoperative score of 17.23/18 (Table 5). A residual LLD of 1 cm was observed after surgery in four patients (25%), compensated by an orthopedic insole. A slight limp was present in 25% of our patients after surgery.

**Table 5:** Summary of the Postel and Merle d'Aubigné score by type of congenital hip dislocation after surgery, with an average follow-up of 22 months The Postel and Merle d'Aubigné score was adapted in our study, as described in previous publications [14].

Types of dislocations	Pain	Mobility	Ability to walk	Total PMA score
Dysplasia	5.87	6	5.43	17.3/18
High non-fixed dislocation (posterior)	5.67	6	5.52	17.19/18
High fixed dislocation (intermediate)	5.76	6	5.23	17.28/18
Low dislocation (anterior)	5.81	6	5.35	17.16/18
The series	5.77	6	5.38	17.23/18

## Discussion

In this retrospective series of 16 patients (19 hips) treated with THA for CHD, we observed a significant improvement in functional outcomes, with the mean PMA score increasing from 8.41 to 17.23. Despite the technical complexity of these procedures, implant stability was achieved in all cases using cemented components, structural bone grafts, and soft tissue releases. However, complications were not uncommon, including intraoperative fractures and nerve injuries, which underscore the surgical challenges specific to THAs in CHDs. The modified Co classification system proved to be a reliable tool for radiographic assessment and surgical planning.

These clinical findings must be interpreted in the context of the unique anatomical and biomechanical challenges posed by CHD. CHD is defined by the presence of the femoral head outside the original acetabulum. This abnormal situation is often accompanied by various anatomical anomalies: hypoplasia of the iliac and femoral bones, abnormal insertion of the capsule on the upper edge of the paleoacetabulum, as well as a change in the architecture, tension, and direction of the periarticular muscles (such as the psoas and gluteal muscles). Additionally, there may be a femoral ascent.

Despite advances in hip surgery, THA for CHD remains very difficult and controversial. Charlney et al. have strongly advised against operating on patients with a late diagnosis of CHD [17]. A comprehensive understanding of this pathology and its complex landmarks, together with rigorous preoperative analysis, is the only guarantee of a good outcome. The imaging assessment of the anatomical changes relies on several classification systems, most of them published in the English literature, such as Crowe and Hartofilakidis [5,8]. According to Crowe et al., hip displacement is classified into four groups based on subluxation, proximal dislocation, and pelvic height ratio. Group I: subluxation < 50%, proximal dislocation < 10%, pelvic height ratio < 0.1. Group II: subluxation 50-75%, proximal dislocation 10-15%, pelvic height ratio 0.1-0.15. Group III: subluxation 75-100%, proximal dislocation 15-20%, pelvic height ratio 0.2. Group IV: subluxation > 100%, proximal dislocation > 20%, pelvic height ratio > 0.2 [8]. 

While many authors use Crowe's classification, it presents notable weaknesses, particularly in the retrospective analysis of pelvic radiographs. Accurate assessment requires visualization of the full extent of the pelvis to correctly measure pelvic height. In addition, identification of the head/neck junction with the teardrop may be challenging. The Hartofilidakis classification divides developmental dysplasia into three categories: dysplasia, low dislocation, and high dislocation [5]. Several authors have suggested that the Hartofilidakis classification may more accurately predict the clinical outcome of THA in CHDs [18].

In our series, we have used the French SoFCOT modified Co classification; its primary advantage lies in its ability to distinguish anomalies with a broader grading scale. According to a study by Clavé et al., in which five French orthopedic experts in hip dysplasia classified 179 hip radiographs, the inter- and intra-observer reproducibility of the modified Co classification system was equal to Crowe’s and Hartofilakidis’s systems [9].

Successful surgical management requires the establishment of a clear benefit-risk agreement with the patient, as well as a realistic expectation-outcome balance. This balance helps address key questions regarding patient expectations, objectives, and optimal timing of the intervention. It is important to consider whether the procedure should be avoided in elderly patients who may be unable to cope with a long and demanding recovery process [6].

**Figure 7:**
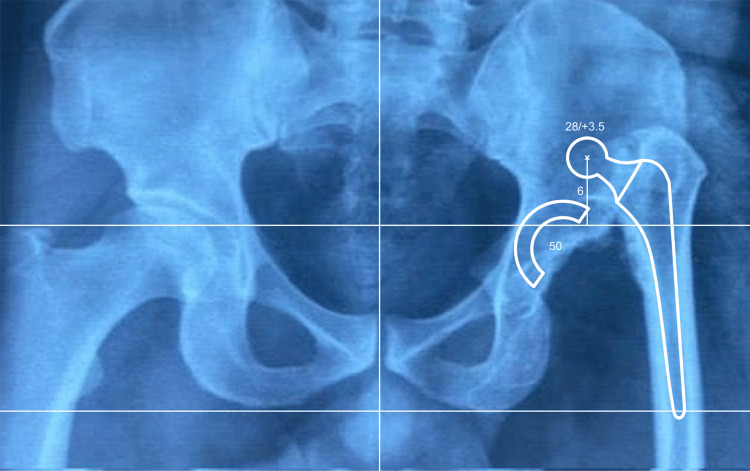
Preoperative planning using overlay templates on an anteroposterior pelvic radiograph of the patient shown in Figure 3, in order to determine the optimal positioning of the femoral stem and acetabular components

Our primary objectives were to achieve a mobile, stable, and pain-free hip; appease associated symptoms (especially spinal and knee discomfort); and restore limb length equality to prevent complications related to nerve involvement. Achieving these goals requires a precise understanding of anatomical abnormalities. A standard radiographic evaluation, including anteroposterior view of the pelvis and lateral view of the hip, is insufficient. A complementary CT scan with 3D reconstruction is essential to evaluate the diameter of the original acetabulum and femoral canal, as well as sections through the knee and foot to reconstruct the anatomical axis of the femur and detect any rotational deformities. Preoperative planning using overlay templates to determine the optimal positioning of the stem, head, and acetabular component is crucial to avoid intraoperative surprises (Figure 7).

The choice of surgical approach in THA for CHD is essential for the proper management of such cases. Both the Moore and Hardinge approaches have limitations and advantages. While Moore provides excellent exposure and access to soft tissues around the hip, it carries a high risk of dislocations and trochanteric issues. The Hardinge approach offers the benefits of preserving soft tissues, reducing the risk of postoperative dislocation, and providing direct access to the acetabulum. Choosing between these two approaches depends on the degree of deformity, trochanteric involvement, and the surgeon's experience [19].

The use of trochanterotomy in THA for CHD remains a subject of ongoing debate. While it can be beneficial in cases of severe deformities and long-standing hip dislocations by providing better access for proper stem positioning, it also carries a high risk of complications, such as trochanteric nonunion, heterotopic ossification, and greater trochanter displacement. Flecher et al. suggest that when performed judiciously and in carefully selected cases, trochanterotomy in THA for CHDs can result in favorable functional outcomes [20]. However, the decision to use this technique should take into account the degree of deformity and the surgeon’s experience.

The paleocotyl represents the anatomical position, the ideal center of rotation of the hip, which allows us to have the optimal biomechanical conditions for the functioning of the limb. On the other hand, the neoacetabulum is an 'in situ' or 'high hip center' insert; the stress on the cup is excessive, the limb is not restored in length, and the adductor muscles are less effective, resulting in poor clinical and radiological results (high risk of loosening and wear of implant material). Nevertheless, THA placement at the neoacetabulum level remains acceptable in elderly or frail patients. A biomechanical study of 640 rotational positions showed that the location of the hip center of rotation (HCOR) after THA is an important factor that could determine the hip joint reaction force (JRF) and the abductor muscle force (AMF) by changing the moment arm of the abductor muscles. This leads to excessive energy consumption and premature wear of the arthroplasty material [21].

During placement of the acetabular cup in the native acetabulum, superior coverage is often insufficient, leaving the cup partially uncovered. In such cases, the use of a structural bone graft secured with screws is generally effective in compensating for the bone defect [6]. According to Goto et al. [22], graft consolidation occurs in approximately 86.4% of these cases. When the acetabular walls are compromised, reinforcement with a Kerboull-type reinforcement cage is recommended to provide additional structural support.

Reaming of the femoral canal should be performed at the slightest uncertainty during femoral stem preparation. Anatomical deformities of the femur in patients with CHD, along with soft tissue variations and LLDs, can make the use of standard femoral stems unsuitable. In such scenarios, custom-made femoral implants may offer a viable solution. These personalized implants are specifically designed to restore normal hip biomechanics and accommodate the abnormal proximal femoral morphology. This approach optimizes physiological stress distribution along the femoral shaft, thereby reducing micromotions and postoperative pain [23].

In our series, the use of cemented components, bone grafts, and soft tissue release techniques ensured satisfactory implant stability and functional restoration. The postoperative improvement in the PMA score (from 8.41 to 17.23 on average) aligns with outcomes reported in similar series in the literature [5]. However, our complication rate (especially intraoperative fractures and nerve injuries) highlights the technical challenges we encounter in these cases. The Co-modified classification proved to be a reliable tool for classifying patients and guiding surgical strategy.

However, this study has multiple limitations that should be acknowledged. First, because it looks back at patient records rather than following patients prospectively, there may be some bias in how cases were selected or how information was recorded. Second, with only 16 patients and 19 hips included, the sample is quite small, which makes it harder to draw conclusions that would apply to a larger population. Third, we relied only on the PMA score to assess functional outcomes. While it’s a well-established tool, it doesn’t reflect all aspects of recovery, such as how satisfied patients were or how the surgery impacted their daily life. We also didn’t have a comparison group, which limits how much we can say about how our results compare to other cases. Finally, the study covers a long period (14 years) during which surgical techniques, implants, and imaging practices have changed, which may have influenced some of the outcomes.

## Conclusions

THA for CHD remains a technically challenging procedure due to complex anatomical deformities like a shallow acetabulum and increased femoral anteversion. These cases require careful planning and a personalized surgical approach to achieve good outcomes. Although patients often experience significant pain relief, full functional recovery is not always possible because of persistent biomechanical issues or muscle imbalance. That’s why ongoing research, better long-term follow-up, and continued refinement of surgical techniques are so important for improving both durability and patient quality of life.
